# The promises and challenges of patient‐derived tumor organoids in drug development and precision oncology

**DOI:** 10.1002/ame2.12077

**Published:** 2019-08-13

**Authors:** Lauren M. Granat, Ooha Kambhampati, Stephanie Klosek, Brian Niedzwecki, Kian Parsa, Dong Zhang

**Affiliations:** ^1^ Department of Biomedical Sciences, College of Osteopathic Medicine New York Institute of Technology Old Westbury New York

**Keywords:** drug testing, patient derived tumor organoids, precision oncology, tumor models

## Abstract

In the era of precision medicine, cancer researchers and oncologists are eagerly searching for more realistic, cost effective, and timely tumor models to aid drug development and precision oncology. Tumor models that can faithfully recapitulate the histological and molecular characteristics of various human tumors will be extremely valuable in increasing the successful rate of oncology drug development and discovering the most efficacious treatment regimen for cancer patients. Two‐dimensional (2D) cultured cancer cell lines, genetically engineered mouse tumor (GEMT) models, and patient‐derived tumor xenograft (PDTX) models have been widely used to investigate the biology of various types of cancers and test the efficacy of oncology drug candidates. However, due to either the failure to faithfully recapitulate the complexity of patient tumors in the case of 2D cultured cancer cells, or high cost and untimely for drug screening and testing in the case of GEMT and PDTX, new tumor models are urgently needed. The recently developed patient‐derived tumor organoids (PDTO) offer great potentials in uncovering novel biology of cancer development, accelerating the discovery of oncology drugs, and individualizing the treatment of cancers. In this review, we will summarize the recent progress in utilizing PDTO for oncology drug discovery. In addition, we will discuss the potentials and limitations of the current PDTO tumor models.

## 1 INTRODUCTION

### 1.1 Cancer cell lines as tumor models

**Figure 1:**
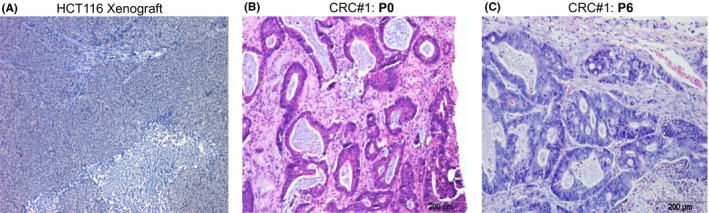
Comparison of tissue hierarchy of cancer cell line and patient‐derived tumor xenograft. A, Hemotoxylin and Eosin staining of xenograft tumors generated from an established colorectal cancer cell line, HCT116. B and C, Hemotoxylin and Eosin staining of colorectal cancer (CRC) patient‐derived tumor xenograft at Passage 0 (P0) and Passage 6 (P6)

Ever since the establishment of the HeLa cell line, which was derived from an African‐American woman with cervical adenocarcinoma and cultured in the 1950s,^1^ 2D cultured cancer cell lines have been instrumental in basic cancer research as well as the development of oncology drugs. However, there are certain limitations of cancer cell lines that must be taken into consideration for both basic cancer research as well as drug discovery.^2^ First, cancer cell lines lack the heterogeneity of primary tumors (Figure 1).^3^ One possible explanation is that only a few types of cells are able to survive the long‐term in vitro 2D culture conditions; therefore, the survived cells are relatively homogeneous in nature.^4^ Second, in vitro 2D culture condition may induce certain genetic alterations, most of which may not be present in cells when grown in vivo.^4^ Third, the growth medium used for culturing cancer cell lines is not able to completely mirror the conditions and environment that tumor cells naturally reside in. In vivo, tumor cells are surrounded by fibroblasts, blood vessels, and immune cells, and their collective interactions are important; this aspect is unfortunately missing in the cultured cancer cell lines.^5^ Therefore, the in vitro cultured 2D cancer cell lines are the least faithful tumor model to be able to recapitulate patient tumors. By growing the established cancer cell lines in a three‐dimensional (3D) environment, which mimics the in vivo extracellular matrix, the so called 3D cell culture moves a step closer to the in vivo tumors.^6^ However, the 3D cell culture still lacks the complex tissue hierarchy comparing to the primary tumors.^6^ Therefore, the 3D cell culture is not ideal for investigating the tumor biology and testing oncology drugs.

### 1.2 Genetically engineered mouse tumor models

Due to the aforementioned limitations, another commonly utilized model in cancer research is the genetically engineered mouse tumor (GEMT) model. In contrast to transplanting cancer cell lines into mice, which requires an immunocompromised status of the host mice to prevent rejection, GEMT is immunocompetent.^7^ Therefore, GEMT can be potentially used for the investigation of immunotherapy. However, mouse tumor models often do not faithfully recapitulate the human cancers. Furthermore, generation of GEMT models can be a time‐ and effort‐consuming process.

### 1.3 Patient‐derived tumor xenograft models

Patient‐derived tumor xenografts (PDTX) can be generated by implanting surgically removed tumors from patients directly into immunodeficient mice. In this model, tumors can be either implanted orthotopically, that is, in the anatomic location of the parental tumor, or heterotopically, that is, in a location unrelated to that of the parental tumor.^8^ This method has certain advantages over the aforementioned cancer cell lines and GEMT. First, the tumor cells can be passaged without the in vitro culture step, thus avoiding the in vitro culture‐induced genetic changes and clonal selection.^9^ Second, the tumors can be implanted alongside their stroma, which may more faithfully mimic the microenvironment of the parental tumors.^10^ While extremely beneficial, the time to develop the PDX model can be long, sometimes taking up to 8 months to develop a single model.^3^ When considering personalized therapeutics, many patients do not have the luxury of such an extended period of time. Lastly, the cost for the development of the PDTX models can be high because immunodeficient mice that the PDTX model requires are very expensive.^5^

### 1.4 In vitro organoids as disease models

Despite the advances that have been made using in vitro cultured cancer cell lines, GEMT, and PDTX, the need remains for more accurate, timely, and less resource‐intensive cancer models. Patient‐derived tumor organoids (PDTO) may be well suited to fit this need.

An organoid is often described as an in vitro generated 3D cellular structure that architecturally and functionally mimics a particular organ/tissue.^11^, ^12^ It can be defined by a few key characteristics, including: (a) self‐organization from stem cells/organ progenitor cells to resemble the 3D in vivo structure; (b) composing of multiple organ‐specific cell types; (c) recapitulating at least some functions of the organ. While the formal use of organoids in research is relatively recent, it is an extension of the continuous efforts to create more accurate representations of in vivo biological processes by modifying in vitro culture conditions.^13^ For example, in 1987, mammary epithelial cells were grown on reconstituted basement membranes instead of on plastic. This new method greatly enhanced the cells’ in vivo‐like morphology and functionality in producing milk proteins.^14^ Despite some advancement, those models are not yet complete enough to be considered in vitro grown organs. Nonetheless, the use of organoids in research has been formalized recently, and research conducted as recently as 2009 investigated organoid development from murine intestinal stem cells.^15^ Sato et al sought to create a culture system, which maintains the physiology of specific in vivo structures within the gut. The system involved stimulating the Lgr5^+^ intestinal cells with relevant gut growth factors in a 3D environment. Such factors included R‐spondin‐1, which enhances Wnt signaling, EGF, noggin, BMP inhibitor and 3D‐Matrigel, which is an artificial laminin‐rich extracellular matrix. The organoids maintained the architecture similar to small intestine, that is, structures that resembled villi and crypts. The genome of these organoids remained remarkably stable over time and through serial passaging, as determined by whole‐genome sequencing of both early and late passaged organoids.

Based on these successes, organoid usage has expanded and this method has been further developed to model various human diseases, in an effort to better understand their molecular mechanisms and to develop therapeutics. One example of this is the use of organoids as models for cystic fibrosis (CF). Dekkers et al^16^ used rectal biopsies of patients with CF to develop organoids for drug testing. CF is a disease that results from a mutation of the CFTR gene, which leads to impaired ion transport and abnormally viscous secretions in the respiratory tract, intestines, pancreas, liver, and reproductive tract.^17^ These CF organoids, due to their histologic and genetic similarities with those of the parental tissue, were able to predict which drugs would work most effectively depending on the specific CFTR mutation the patient was harboring.^16^ The CF organoids have also allowed investigators to measure levels of CFTR functionality to develop patient‐specific therapies. Patient‐derived organoids have also been utilized as models for a variety of other genetic disorders, including two liver disorders, alpha 1‐antitrypsin deficiency (A1ATD) and Alagile syndrome.^18^ In both instances, the histological and mutational characteristics of the organoids closely mirrored those of the patients, rendering this an effective model for elucidating specific mechanistic details and developing novel treatment modalities.

Another important application of patient‐derived organoids, and the primary focus of this review, is in their utility within oncology research and drug discovery. Furthering the advances made in growing organoids from healthy intestinal tissue and from patients with various genetic disorders, researchers were also able to successfully establish organoids from patients with many different types of cancer, that is, the PDTOs. We have thoroughly searched the literature using the key words “organoids”, “drugs”, and "drug testing”. The utility of PDTOs in modeling various types of cancers and in serving as drug screening tools will be the focus of this review. We will discuss the utility of PDTOs in drug testing in the following malignancies: prostate adenocarcinoma, breast carcinoma, pancreatic adenocarcinoma, gastric adenocarcinoma, metastatic gastrointestinal cancer, hepatocellular carcinoma, esophageal adenocarcinoma, urothelial carcinoma, endometrial adenocarcinoma, mesothelioma, and appendiceal carcinoma (Table 1). We think that PDTOs have the potential to serve as models for pretreatment screenings for cancer patients and to increase the successful rate of oncology drug development.

**Table 1:** Drugs tested using the PDTOs

Cancer type	Success rate of PDTO	Drugs tested	Reference
Prostate adenocarcinoma	18% (6/32)	Enzalutamide, Everolimus, BKM‐120	Gao et al^19^
Breast carcinoma	~80% (>155)	Afatinib, Pictilisib, Everolimus, Olaparib, Niraparib, Tamoxifen	Sachs et al^20^
Pancreatic adenocarcinoma	75% (103/138) 85%(17/20)	FOLFIRINOX (5‐ Fluorouracil, leucovorin, irinotecan, oxaliplatin), Gemcitabine, Paclitaxel, SN‐38 Gemcitabine, UNC1999	Tiriac et al^21^ Huang et al^22^
Gastric adenocarcinoma	71% (10/14)	Cisplatin, Irinotecan, Oxaliplatin, 5‐Fluorouracil	Gao et al, 2018^23^
Metastatic gastrointestinal carcinoma	70% (>100) 76% (13/17)	Paclitaxel, Cetuximab, Regorafenib, TAS‐102 Oxaliplatin, Capecitabine, 5‐Fluorouracil	Vlachogiannis et al^24^ Buzzelli et al^25^
Hepatocellular carcinoma	26% (10/38) 100% (8/8)	Sorafenib Taselisib, Gemcitabine, AZD8931, SCH772984, Dasatinib	Nuciforo et al^26^ Broutier et al^27^
Esophageal adenocarcinoma	31% (10/32)	5‐Fluorouracil, Epicubicin, Cisplatin	Li et al^28^
Urothelial carcinoma	70% (12/17)	Over 20 compounds, including: Trametinib, SCH772984	Lee et al^29^
Endometrial carcinoma	100% (15/15)	BB1608, Paclitaxel, Cisplatin, Tyrosine Kinase Inhibitors, Fulvestrant, Megestrol Acetate, Medroxyprogesterone Acetate, Levonorgestrel	Girda et al^30^
Mesothelioma	100% (2/2)	Cisplatin‐Pemetrexed, Carboplatin‐Pemetrexed	Mazzocchi et al^31^
Appendiceal carcinoma	75% (9/12)	5‐Fluorouracil, Oxaliplatin, FOLFOX (5‐Fluorouracil, Oxaliplatin, Leucovorin), FOLFIRI (5‐Fluorouracil, Irinotecan, Leucovorin, Regorafenib, Pembrolizumab, Nivolumab)	Votanopoulos et al^32^

## 2 PDTOS AS POTENTIAL TUMOR MODELS FOR DRUG SCREENING AND DRUG DEVELOPMENT

### 2.1 Prostate adenocarcinoma‐derived tumor organoids

Prostate cancer (PC) is the most common malignancy, and the second most common cause of cancer‐related deaths, in men in the United States.^33^ Recent research has shown great success with antiandrogen therapies, yet the current disease models have made in vitro investigations of PC very difficult. Gao et al^19^ modeled advanced PC using organoid cultures. The research team sought to create an organoid model that mirrored the patients’ tumors, both histologically and genetically, and that could be utilized in drug testing.

The research team plated metastatic PC samples collected via both bone and soft tissue biopsy and successfully propagated 6 out of 32 for more than 6 months, yielding a success rate of approximately 15%‐20%. A seventh organoid line was generated from circulating PC tumor cells of a castration‐resistant prostate cancer (CRPC) patient. The tumor samples that were not successfully propagated were able to be maintained for 1‐2 months, but were eventually overtaken by tumor‐associated spindle cells or normal epithelial cells present in the biopsy material.

Copy number analyses were conducted on the organoids to determine their similarity to the profiles of the parental tumors. It was found that three of the PC organoids contained homozygous deletions of the gene encoding chromodomain helicase DNA binding protein 1 (CHD1), which is the second most commonly deleted gene in prostate cancer. In addition, six of the seven PC organoid lines contained homozygous deletions of PTEN, and one of the PC organoids harbored an amplification of the gene encoding androgen receptor (AR), which is found in approximately 50% of CRPC.

Whole‐exome sequencing was also conducted on the PC organoid lines, and the results suggested that the mutational profiles of the PC organoids were consistent with those of prostate cancers as a whole. Four PC organoids had mutations in TP53 gene, which is the most commonly mutated gene in CRPC. The PC organoid that was derived from circulating tumor cells, rather than the metastatic biopsies, harbored a mutation in the SPOP gene, which encodes an E3 ubiquitin ligase and is the most commonly mutated gene in primary prostate cancers. Mutational profiles of the PC organoids were also compared to those of the parental tumors, and it was found that there was a high concordance between the allele frequencies found in organoid lines and the corresponding tumor samples. Histological analyses revealed that the PC organoids largely retained morphological features of the primary tumor, despite the fact that, in some cases, the morphological features of the metastasis differed.

Due to the relative similarity of the PC organoids to their parental tumors, the research team performed growth assays to determine the sensitivity of the PC organoids to enzalutamide, an antiandrogen, and two PI3K pathway inhibitors, everolimus and BKM‐120. The PC organoid that harbored an AR amplification was extremely sensitive to enzalutamide, with an IC‐50 of approximately 50 nmol/L, in comparison with the PC organoids that were lacking this amplification and were, as expected, resistant to this agent. These results were further confirmed when the PC organoids were transplanted as xenografts, and the AR‐amplified PC organoid displayed sensitivity to enzalutamide in this setting as well. It was also found that the PC organoid line, which harbored both the PTEN and PIK3R1 mutations, was sensitive to everolimus and BKM‐120, in keeping with the targets of these drugs.

Collectively, these results show that PC organoids can be a useful model for investigating aggressive prostate cancer, and for the therapeutics that have been designed to target specific subsets of the disease. Since taking biopsies and characterizing the metastatic lesions has become increasingly common practice, a biobank for the metastatic PC organoids can be developed. Utilizing the biobanked PC organoids with specific genetic profiles for drug testing will be crucial in devising a more personalized treatment regimen for prostate cancer patients.

### 2.2 Breast carcinoma‐derived tumor organoids

Breast cancer (BC) is the most common malignancy among females in the United States, and the second leading cause of cancer‐related mortality in this cohort.^33^ Carcinoma of the breast can be classified as either carcinoma in situ or invasive carcinoma. Carcinomas in situ can be further divided into either the ductal or lobular subtype, and invasive breast carcinomas are characterized via various histologic findings.

In a recent study conducted by Sachs et al,^20^ a protocol was developed to successfully establish more than 100 primary and metastatic BC organoid lines with an 80% success rate. The BC organoids were distributed randomly across the major subtypes of breast cancers. While the BC organoid histology could not completely mirror tissue histology due to a lack of mesenchymal cells and other in vivo factors, there was moderate consistency between the parental tumor histologic appearance and the organoid appearance. For example, ductal subtypes more frequently gave rise to a solid organoid, while lobular tumor subtypes gave rise to discohesive organoids. This was verified by performing a blinded tissue and organoid analysis. The expression status of ER/PR/HER2 in BC organoids and their parental tumors were also largely matched. The organoid histological analyses often correlated with the parental tumor status (eg, well‐differentiated vs poorly‐differentiated). However, some of the more well‐differentiated tumors were mistaken for normal tissues, suggesting that the more subtle malignancies could be more difficult to detect by this type of analyses. The BC organoids maintained copy number alterations (with cleaner, more distinct copy number signals found in the organoids, compared to those in the parental tumor) and sequence changes were maintained over extended passaging.

Next, Sachs et al evaluated whether the BC organoids could be used for drug screening. They compared the response of BC organoids to certain HER2 pathway blockers, including Afatinib, Pictilisib, and Everolimus, and PARP inhibitors (PARPi), such as Olaparib and Niraparib. In the majority of the BC organiods, response to the HER2 pathway blockers largely correlated with the overexpression of HER2. However, a few BC organoids did defy the HER2 correlations. The reason for this inconsistency is unclear. Similarly, the mutant BRCA1/2 signature of the BC organoids correlated well with their sensitivity to PARPi. Most intriguingly, by comparing the 12 BC organoids established from needle biopsies of patients with metastatic BC, they observed similar response to tamoxifen, suggesting that BC organoids may potentially be used to predict the drug's response of BC patients in the clinic.

Similar to the PC organoids, it is also feasible to establish a biobank of BC organoids. On the one hand, the BC organoid biobank will preserve the patient samples long‐term. On the other hand, once the genetic and epigenetic profiles of the BC organoids are thoroughly characterized, they can be used for drug screening to develop drugs that target a particular BC profile.

### 2.3 Pancreatic adenocarcinoma‐derived tumor organoids

Pancreatic ductal adenocarcinoma (PDAC) is one of the most deadly cancers in the US.^33^ Patients with pancreatic cancer have very short mean survival times, partly due to the fact that many patients present without symptoms, or may only present with symptoms at a very late stage. Therefore, methods that allow for the rapid determination of individualized therapies are crucial in improving the survival rate of these patients.

Tiriac et al^21^ conducted a study to compare chemotherapy responses of PDAC patient‐derived organoids to the parental tumors. Among the 69 PDAC organoids with patient clinical data available, 96% had KRAS mutation, and 88% harbored TP53 mutations as well as numerous mutations in genes like CDKN2A and SMAD4. On average, 97.43% of the mutations that were detected in the primary tumor specimen were also detected in the organoids. Copy number analyses of the paired primary tumors and their respective organoids showed concordance with high purity; however, most primary tumor specimens had insufficient purity to reveal copy number alterations.

The PDAC organoids were able to recapitulate patient response to the most commonly used chemotherapeutic agents in this cohort. Of the six patients who had progression‐free survival longer than the mean survival time, five of them were treated with at least one drug to which the matched PDAC organoids were also particularly sensitive. Of the three patients who rapidly progressed, two were treated with a chemotherapeutic agent to which their PDAC organoids were markedly resistant.

The PDAC organoids were also able to reflect the temporal evolution of an individual patient for whom extensive longitudinal data were available. A PDAC organoid was isolated from the resection of lung metastasis, which was sensitive to FOLFIRINOX (5‐fluorouracil, leucovorin, irinotecan, and oxaliplatin) and a combination of Gemcitabine and Paclitaxel. This was in line with the clinical response of the patient. Two years later, the patient presented with progressive disease that histologically resembled neuroendocrine, small‐cell lung cancer and died shortly after. PDAC organoids that were derived from the neuroendocrine tumor postautopsy displayed amplification of KRAS and were resistant to Gemcitabine, Paclitaxel, SN‐38 and possessed a basal‐like histological pattern. This finding indicates that the organoids were able to mirror both positive and negative chemotherapeutic responses.

The library of PDAC organoids was also used in determining the efficacy of various targeted therapies among specimens that were resistant to the commonly used chemotherapeutic agents. Of the PDAC organoids that lacked sensitivity to the aforementioned agents, 21 organoids were then tested with targeted agents. The authors were able to identify targeted agents with extreme PC organoids sensitivity for half (n = 11), including the kinase inhibitor Sunitinib.

Lastly, transcriptional analyses were conducted to generate drug signatures that would then be correlated with drug response, as measured by the AUC of individual patients undergoing clinical trials. It was found that, among the patients who responded to Gemcitabine, 50% had the transcriptional signature, and that those who possessed the signature had higher rates of progression‐free survival. In addition, patients who responded to Oxaliplatin had PDAC organoids that displayed its respective drug signature. However, this pattern was not seen with regard to 5‐FU and SN‐38.

In a separate study, Huang et al^22^ also sought to model PDAC using organoids, and did so via samples derived from both human pluripotent stem cells (PSC) and patient tumors. The authors utilized FGF2, insulin, hydrocortisone, ascorbic acid, all‐trans‐retinoic acid, and B27 serum‐free supplement in their culture medium and were able to successfully create 3D structures from approximately 15% of PSC‐derived cells. While these organoids displayed general pancreatic features, further differentiation into pancreatic ductal cells, specifically, was needed to faithfully recapitulate ductal pancreatic cancer models. Wnt, Notch, and TGFB growth factors were utilized, and these factors were able to induce differentiation of the organoid lines.

The research team also investigated whether or not the cultured organoids could serve as models for phenotypes associated with the two most frequently seen mutations in ductal pancreatic cancer, mutant KRAS and TP53. Both mutant KRAS and TP53‐infected cells had higher proliferation rates compared to the negative control. Further, both KRAS and TP53‐mutated organoids displayed disorganized morphology consistent with the in vivo findings.

The research team also generated organoids from PDAC patient tumor samples, and was able to successfully propagate 17 of 20, yielding a success rate of 85%. Histologically, the PDAC organoids retained similar features as the primary tumors, including the expression of differentiation markers such as KRT19, GATA6, and SOX9. These organoids were transplanted into immunodeficient mice and maintained morphological features of the parental tumor as well.

Finally, the research team conducted drug testing on the PDAC organoids, and found that the organoid lines did not respond well to gemcitabine, despite the fact that the parental tumors had a significant response to this standard of care therapeutic. However, positive results were found when targeted therapies were utilized, and organoids and parental tumors alike that harbored the EZH2 mutation responded well to UNC1999, an inhibitor of EZH2, when combined with gemcitabine.

The results from the aforementioned studies indicate that PDAC organoids can be developed from both pluripotent stem cells and patient tumor samples. These organoids were able to faithfully recapitulate the genetic and morphological features of PDAC. Once biobanked, the PADC organoids will serve as valid models for developing personalized therapy for PDAC.

### 2.4 Gastric adenocarcinoma‐derived tumor organoids

There are two main types of gastric adenocarcinoma: the intestinal subtype, typically associated with *Helicobacter pylori* infections, and the diffuse subtype. Due to late presentation and vague symptoms, gastric adenocarcinoma is often diagnosed at late stages, and the survival rates are typically low.^34^ As a result, timely and effective models for gastric adenocarcinoma are urgently needed.

Gao et al^23^ investigated the utility of patient‐derived organoids in this population. Samples were retrieved from esophagogastroduodenal (EGD) biopsies, which patients typically undergo at the time of diagnosis and early staging. Due to concerns about the ability of organoids derived from endoscopic biopsies to adequately capture the parental tumor's heterogeneity, organoids were also derived from surgical gastrectomy samples, and histological and genetic characteristics were compared between both cohorts. The authors hypothesized that organoids generated from EGD biopsies would recapitulate features of both the surgically derived organoids and the parental tumors, in hopes that the EGD method would become widely used due to its low‐risk profile and relative ease.

Using immunofluorescent staining, it was determined that the endoscopy‐derived organoids expressed both LGR5 and TROY, two gastric epithelial markers, indicating their gastric origin. Whole‐genome profiling of the paired EGD and surgical organoids, as well as the whole‐tumor lysates, demonstrated fewer copy number variation. PCR assays identified similar KRAS alterations in the primary tumor and paired organoids. This finding highlights the ability of this method to maintain genetic alterations from primary tumor to both surgical and endoscopic organoids.

The PDOs from both surgical and EGD cohorts responded similarly to agents typically used to treat gastric adenocarcinoma: cisplatin, irinotecan, and oxaliplatin. It was found that these agents lead to cytotoxicity in both subsets of organoids, again confirming the utility of EGD‐derived organoids. In addition, one patient was treated with 5‐fluorouracil (5‐FU) after organoid generation, and therefore the organoid drug response was able to be compared to the clinical findings. Organoids from this patient were treated with 5‐FU, and their response was largely correlated with the positive clinical response. Due to the success of the EGD‐method of generating gastric cancer organoids, future studies should focus on more extensive genetic analyses, which would lay the groundwork for personalized drug screening.

### 2.5 Metastatic gastrointestinal carcinoma‐derived tumor organoids

Colorectal carcinoma (CRC) is the third most common malignancy in the United States among both females and males, and is also the third most common cause of malignancy‐associated deaths.^33^ The most common site for CRC to metastasize to is the liver, and liver metastases are a poor prognostic factor.^35^ Current cell lines do not accurately represent advanced disease and have not proven to be helpful for modeling metastatic CRC.^25^

A recent study conducted by Vlachogiannis et al^24^ sought to compare the drug responses of patient‐derived organoids to those of the metastatic gastrointestinal cancer patients via a coclinical trial. The study analyzed 110 organoids, which were derived from biopsies of 71 metastatic gastrointestinal cancer patients. The patients in the study cohort had one of the following tumors: metastatic CRC, metastatic cholangiocarcinoma, or metastatic gastric carcinoma. The patients were enrolled in one of four clinical trials, either phase I or II. The drug responses of the organoids were compared to the drug responses of the patients while enrolled in the clinical trial.

The authors first confirmed the validity of using organoids as tumor models via analyzing histology, immunohistochemistry, genome sequencing, copy number alterations, and 3‐day drug screening assays. Drug responsiveness testing was conducted with the following targeted agents and chemotherapy drugs: paclitaxel, cetuximab, regorafenib, and TAS‐102. It was found that the organoids' ability to predict clinical response to targeted agents and chemotherapy drugs had a 100% sensitivity, 93% specificity, 88% PPV, and 100% NPV.

In a separate study, Buzzelli et al^25^ assessed the validity of organoids derived from liver metastases of CRC. Seventeen samples were taken from patients who met the inclusion criteria, and 13 organoids were developed, with a 76% success rate. The patients included had either stage T3 or T4 CRC and had received three to six cycles of chemotherapy.

There are many tumor markers that are used as indicators of the advanced disease in metastatic CRC and are frequently used to assess prognosis. Specifically, EpCAM, MUC2, and CEACAM1 were used to determine if the developed organoids could recapitulate disease in patients with advanced metastatic CRC. It was found that the expression pattern of MUC2, CEACAM1, and EpCAM in the CRC organoids corresponded to those of the patients, and demonstrated that the colorectal cancer liver metastases organoids show characteristics of advanced stage disease.

All of the patients included in the study had been first treated with Oxaliplatin and Capecitabine, and due to their current advanced disease status, resistance to these therapies was assumed. To determine if these organoids displayed similar drug resistance patterns to their parental tumors, morphology and growth patterns were assessed after multiple rounds of chemotherapy. The most noticeable change in morphology after multiple rounds of 5‐FU and Oxaliplatin was found after three rounds and resulted in the loss of distinct lumen‐like structures. The organoids acquired resistance to 5‐FU and Oxaliplatin in similar patterns to the patients. One explanation for this finding is that both 5‐FU and Oxaliplatin preferentially target proliferating cells, therefore it is likely that the significant growth delay in colorectal carcinoma liver metastasis organoids following multiple rounds of chemotherapy allowed the organoids to evade chemotherapy.

Taken together, these recent studies demonstrate that it is possible to establish a metastatic gastrointestinal carcinoma organoid biobank. These organoids should preserve the patients’ tumors quite well and therefore can be used to develop better treatment strategies for the metastatic gastrointestinal cancer patients.

### 2.6 Hepatocellular carcinoma‐derived tumor organoids

Hepatocellular carcinoma (HCC) is a malignancy of the liver, and is typically a sequelae of chronic liver diseases, such as cirrhosis. Risk factors for developing this malignancy include: chronic hepatitis B and C infections, exposure to aflatoxin, alcohol abuse, and tobacco use.^36^

A recent study conducted by Nuciforo et al^26^ investigated the utility of organoids in modeling HCC. The research team collected tumor and nontumor liver samples via ultrasound‐guided needle biopsies. This methodology allowed for the collection of up to five samples from the same location, and is preferred over collection via surgical resection due to its selection for early stage tumors and noncirrhotic livers. The research team was able to generate HCC organoids from 10 out of 38 tumor specimens, yielding a 26% success rate. While the success rate may seem low at first glance, the authors presume that this was because HCC organoids are more easily generated from high‐grade and poorly differentiated tumors. In fact, all of the tumors that were successfully converted to HCC organoids were poorly differentiated, and were categorized as Edmonson Grade III and IV HCC.

Extensive testing was conducted in an attempt to characterize these HCC organoids, and to determine if they faithfully recapitulated the mutational and histological characteristics of their parental tumors. The HCC organoids maintained the growth pattern and differentiation grade of its primary tumor. It was also found that levels of alpha fetoprotein (AFP), a serological marker commonly seen in hepatocellular carcinoma,^37^ was maintained in both HCC organoids and parental tumors. Other markers were seen in both cohorts as well, including: glypican 3, glutamine synthetase, and heat shock protein 70. Additionally, it was determined that the HCC organoids retained the somatic genetic alterations of the parental tumor, and that while there are new genetic alterations that were seen in only the HCC organoids, it was likely that these mutations were actually present in the parental tumors, albeit at very low frequencies. The HCC organoids were then transplanted into immunodeficient mice as xenografts, and 60% (6 of 10) propagated successfully. All transplanted HCC organoids gave rise to xenograft models that also mirrored the histological characteristics of the parental tumor, as well as maintained serum marker levels.

Drug testing was conducted with sorafenib, a tyrosine kinase inhibitor. Treatment with this agent led to reduced growth of the HC organoids in a dose‐dependent manner. However, matched clinical response was unfortunately unavailable because the patients were not treated with sorafenib.

In a separate study, Broutier et al^27^ generated organoids from eight liver cancer patients, including the three most common subtypes: hepatocellular carcinoma (HCC), cholangiocarcinoma (CC), and combined HCC/CC (CHC) tumors. The research team also noted that it is easier to derive organoids from high‐grade tumors than from low‐grade and well‐differentiated tumors. They also found that the liver cancer organoids were able to mirror both the histological and genetic characteristics of their parental tumors, and therefore may serve as vehicles for drug testing.

It was determined that, in contrast with the healthy liver‐derived organoids, the HCC organoids formed classically seen pseudoglandular rosettes, and the CC organoids formed extensive glandular structures. Biomarker analyses were conducted, which showed that the expression of EpCAM and AFP was seen in CC and HCC, respectively, and that the levels of each mirrored those of the parental tumors.

Whole‐exome sequencing was conducted on the organoid samples, and the research team observed that approximately 80% of genetic variants in the patient's tissue were retained in the organoids after months of expansion. Next‐generation sequencing studies were also conducted, which found mutations that corresponded with the parental tissue, for example CTNNB1 missense mutations in HCC, TP53 frameshift mutations in CHC, and KRAS mutations in both CC and CHC. The authors also compared the transcriptomes of the tumor organoids to those of the healthy‐liver derived organoids, and identified 30 genes that were upregulated in the tumor organoids, some novel and some of which have been implicated in liver cancer in the past. Furthermore, four novel gene signatures were found to be associated with poor survival: C19ORF48, UBE2S, DYTMK (for HCC), and C1QBP (for CC). Further research is warranted to determine the utility of these biomarkers in predicting disease severity and progression.

Drug testing was performed on the tumor organoids utilizing the following clinically relevant compounds: Taselisib, Gemcitabine, AZD8931, SCH772984, and Dasatinib. While most organoids were resistant to the majority of the drugs, a correlation was found between drug sensitivities and mutational profiles of the organoids. Interestingly, the research team found that treatment with SCH772984, an ERK1/2 inhibitor, leads to significant suppression of organoid formation via selectively inhibiting ERK‐phosphorylation in HCC and CC organoids. Of note, these organoids were insensitive to BRAF and MEK inhibitors. To further test these findings, HCC and CC organoids were transplanting into NSG mice. This finding was recapitulated in vivo, and a significant reduction in tumor xenograft growth was noted, likely also due to inhibited ERK‐phosphorylation, as confirmed by Western blot analysis. Further testing is warranted to determine the utility of this therapeutic in the clinical setting.

The results discussed above indicate that liver cancer organoids were able to recapitulate the histologic and genetic features of liver cancers and may serve as useful models for drug screening and development.^26^, ^27^

### 2.7 Esophageal adenocarcinoma‐derived tumor organoids

Esophageal adenocarcinoma (EAC) is an especially aggressive type of cancer, and has a 5‐year survival rate of approximately 15%. EAC is typically the result of long‐standing and untreated gastroesophageal reflux. Investigators and clinicians working with EAC have faced various challenges with developing accurate disease models, due to the complexity of their molecular genetics. Li et al^28^ conducted a study evaluating the utility of patient‐derived organoids in modeling EAC. The authors sought to develop a biobank of organoids that could more accurately depict the molecular heterogeneity of EAC.

The authors successfully generated 10 organoids out of 32 EAC patients with a success rate of 31%. The majority of the EAC organoids were derived from chemo‐resistant donors with advanced stage disease. To determine the organoid's ability to recapitulate various aspects of the EAC, extensive histological and mutational characterizations were performed. To confirm their epithelial origin, the organoids, as predicted, were stained positive for pan‐cytokeratin and negative for vimentin. Eighty percentage of the organoids showed TP53 mutations. The main esophageal adenocarcinoma driver mutations (CDKN2A, KCNQ3, and PIK3CA) were also maintained in the organoids. However, a higher frequency of driver mutations was found in organoids than in the parental tumors, possibly due to the pure tumor cellularity of the organoids. Finally, the dominant mutational signature was similar between the parental tumors and the organoids.

To test the stability of the EAC organoids, 4 of the 10 organoids were propagated and subjected to whole‐genome sequencing at multiple passages over a 6‐month period. The organoid genomes were relatively stable, showing less than 25% increase in the total number of mutations, none of which influenced the various cancer drivers.

Because of the relative stability of the EAC organoids, drug testing was performed using these organoids to standard EAC chemotherapy agents: 5‐FU, epirubicin, and cisplatin. Six of the eight organoids tested were resistant to chemotherapy, which was consistent with the patients’ poor response in the clinic.

### 2.8 Urothelial carcinoma‐derived tumor organoids

Bladder cancer is the fourth most prevalent cancer in men in the United States; however, it is relatively understudied.^33^ The most common form of bladder cancer is urothelial carcinoma, which can be further subcategorized into nonmuscle‐invasive and muscle‐invasive carcinomas. While nonmuscle invasive bladder cancer has a relatively favorable prognosis, muscle‐invasive bladder cancer generally has poor survival rates. Muscle‐invasive bladder cancer can be further categorized into a luminal‐like subtype, and a more aggressive subtype, with basal‐like features.^38^ Treatment options for bladder cancers include multiple trans‐urothelial resections and/or chemotherapy.

A recent study conducted by Lee et al^29^ evaluated the utility of organoids in modeling bladder cancer. Lee et al generated and characterized 22 patient‐derived bladder cancer organoids from specimens obtained from transurethral resections. The organoids were derived from both muscle‐invasive and nonmuscle invasive bladder cancers, and the epidemiological characteristics of the cohort utilized mirror that of the larger cohort of patients with this disease. Since many patients with bladder cancers undergo multiple biopsies throughout their disease course, this allowed the investigators to derive chronologically distinct organoids from the same patient. These unique sets of organoids allowed them to monitor the progression of the tumor. Subsequently, 83% of these organoids were orthotopically implanted into immunodeficient mice as bladder cancer xenografts.

Histological analyses of the bladder cancer organoids, xenografts, and parental tumors revealed striking similarities among them. Various methods of genome sequencing were performed on the bladder cancer organoids and it was found that the organoids displayed clonal evolution during serial passages, which correlate with the changes in vivo as well. Mutational profiles were largely maintained in the organoids. For example, the organoids contained mutations most commonly seen in nonmuscle‐invasive bladder cancers, including ARID1A, KMT2C, KMT2D, KDM6A, and FGFR3. Interestingly, only 33% of the organoids derived from muscle‐invasive bladder cancers harbored TP53 mutations, compared to the reported frequency of 50% in this subpopulation. The rates of Rb mutations in organoids were lower than patient tumors as well. The reason for these discrepancies is unclear.

Phenotypic analyses using immunofluorescence assays were also conducted on the organoids and it was found that 36% of the organoid lines are quite stable. In contrast, 64% (n = 14/22) of the organoid lines differed substantially from their parental tumors. The majority (86%, n = 12/14) of these organoids displayed a shift from the luminal phenotype, which was observed in the parental tumor, to the basal phenotype. Intriguingly, once the basal organoids were implanted as orthotopic xenografts, they reverted back to the luminal phenotype.

Because the bladder cancer organoids are quite similar to their parental tumors, genetically and phenotypically, the authors then tested their response to 50 clinically relevant compounds/drugs, including the standard‐of‐care drugs as well as drugs that are being tested in clinical trials, using 20 different organoids. The results showed that the drug sensitivity partially correlate with the genetic profile of different organoids. For example, two organoids that are sensitive to the treatment of MEK inhibitor trametinib and ERK inhibitor SCH772984 also contain activating mutations in the FGFR3 gene. They also validated the drug response of the cultured organoid using orthotopic xenografts of the same organoid. The results showed that indeed the drug response of the xenograft largely recapitulates that of the cultured organoid.

Finally, the authors examined the drug response of the organoid lines established from the same patient at different times of their disease progression. The drug sensitivity of the organoids seems to track the drug sensitivity of the patient in the clinic, suggesting that the organoids could potentially be used for selecting the best treatment regimen for patients in the clinic.

### 2.9 Endometrial adenocarcinoma‐derived tumor organoids

The most common form of uterine cancer is adenocarcinoma of the endometrium, or the innermost lining of the uterus. The majority of cases of endometrial carcinoma are due to prolonged and high levels of estrogen exposure, and are considered endometrioid in morphology.^39^

Girda et al^30^ generated 15 organoids from 14 endometrial and 1 metastatic tumors. The organoids had similar morphological features to their parental tumors. Immunohistological staining was conducted. The results showed that all grade 1 endometrioid carcinomas expressed estrogen receptors (ER) and progesterone receptors (PR), and that this pattern was maintained in the organoid cultures. However, the Ki‐67 proliferation index and the levels of ALDH1 and CD44 were not always consistent between organoids and their respective tumors.

Most organoids were inhibited by BB1608, a STAT3 inhibitor, suggesting that stem cells were implicated in the growth of the organoids. 78.6% of organoids were inhibited by paclitaxel, a commonly used chemotherapeutic agent for the endometrial carcinoma. However, none of the organoids responded to cisplatin, another commonly used drug in this population. The growth of organoids was significantly inhibited by multiple tyrosine kinase inhibitors. Fifty‐seven percentage of the grade 1 organoids were inhibited by fulvestrant, an estrogen receptor degrader, which suggests the importance of estrogen in endometrial carcinoma organoid development. Response to progestins, including megestrol acetate, medroxyprogesterone acetate and levonorgestrel, was poor among the organoid lines.

Despite the utility of these findings, the authors did not conduct genomic sequencing to confirm the mutational profile of the organoids in comparison to their parental tumors. Future studies should examine the genetic makeup of these models, and investigate their utility in targeted therapy research. In addition, the organoids were analyzed at their first passage only, and this may limit their ability to recapitulate the tumor heterogeneity that results from multiple passages. Finally, patient data would have been helpful to consider alongside the aforementioned results, to determine if the limited results seen with progestins were also seen in the clinical setting.

### 2.10 Mesothelioma‐derived tumor organoids

Mesothelioma is a malignancy of mesothelial surfaces, including the pleura, which is the most common location, peritoneum, pericardium, or tunica vaginalis of the testes.^40^ Mazzocchi et al^31^ conducted a study on mesothelioma organoids derived from two patients (Patient #1 and #2) with the epithelioid subtype of peritoneal mesothelioma and investigated their utility in drug testing.

The two mesothelioma organoid lines, MO #1 and MO #2 retrieved from Patient #1 and #2 respectively, were analyzed extensively via histological methods to assess for viability and the presence of various mesothelioma biomarkers. The research team was able to confirm high cell viability in the organoids over the course of 14 days. Additional histological analyses confirmed that both organoids retained the following mesothelioma biomarkers: CK5/6, a high‐molecular weight keratin, calretinin, a calcium‐binding protein, and thrombomodulin, or CD141.

The histological analysis validated the mesothelioma organoids as faithful models of the patients’ tumors, therefore, the organoids were utilized in subsequent drug testing. First, the organoids were treated with the same chemotherapeutic agents as their parental tumors. Patient #1 was treated with six rounds of cisplatin‐pemetrexed, followed by a single round of carboplatin‐pemetrexed. This treatment regimen lead to a significant clinical response, and the patient had near complete resolution of malignant ascites. Patient #2 was treated with four cycles of cisplatin and pemetrexed with a mixed response. Both mesothelioma organoids were treated with combinations of both cisplatin and pemetrexed and carboplatin and pemetrexed. Organoid responses to these regimens mirrored that of the patients—MO#1 responded well to cisplatin and pemetrexed, while MO#2 did not respond significantly to cisplatin‐based therapy.

Although only two patients were analyzed, the results did indicate that the mesothelioma organoid technology is useful in recapitulating the histological and mutational features of peritoneal mesothelioma. This study lays important groundwork for future studies and for the utilization of organoids in personalized medicine in the field of mesothelioma.

### 2.11 Appendiceal carcinoma‐derived tumor organoids

Primary appendiceal cancers are quite rare, and are estimated to affect approximately 1000 people each year in the United States. It is often difficult for researchers to study rare diseases, as cell lines are sparse and clinical outcomes are typically derived from heterogeneous cohorts. Further complicating the study of appendiceal cancer is that low‐grade appendiceal tumors (LGA) are difficult to culture due to their low cellularity. Votanopoulos et al^32^ sought to overcome these limitations by successfully deriving nine appendiceal tumor organoids from 12 patients, three with high‐grade appendiceal (HGA) tumors and six with LGA tumors. 

Viability and histological analyses were conducted on the nine organoids, and it was determined that the HGA organoids formed more tissue‐like high cell density structures, whereas the LGA organoids were comprised of cells that are more spread out. The research team proposed that this discrepancy was due to the fact that HGA tumors are more metabolically active, and therefore were able to reorganize into complex structures and deposit an extracellular matrix. Immunohistostaining revealed that both HGA and LGA organoids stained positive for MUC2 and CK20, common biomarkers of appendiceal tumor cells.

Chemotherapy sensitivity studies were conducted on therapies commonly utilized in appendiceal cancer, including: 5‐FU, oxaliplatin, FOLFOX, FOLFIRI, and regorafenib. Viability assays indicated that HGA organoids displayed variable responses to these agents, whereas LGA tumors did not respond to any therapy, likely due to their slow cycling. This finding is consistent with the clinical response of patients with LGA tumors. This trend was further confirmed using the mitochondrial quantification assays, and HGA organoids displayed variable responses and LGA organoids completely lacked susceptibility.

The organoids were also subjected to immunotherapy testing, to determine if this modeling system can support immunotherapy‐based drug screens. The research team injected cells from a dissociated lymph node, which was obtained from the same patient at the same time the LGA tumor specimen was obtained, into LGA organoids. Screening was conducted with pembrolizumab and nivolumab, inhibitors of the programmed cell death receptor of lymphocytes. It was found that the organoids that were enriched with lymph nodes displayed signs of activation of T cells, indicating that these models may potentially be used for immunotherapy screening as well.

## 3 CONCLUSION

Personalized medicine offers great promise to the field of oncology, and faithful and timely models of tumors are crucial to the development of personalized therapy for cancer patients. Even though the use of organoids in research has only been formalized 10 years ago,^15^ organoid research has generated much excitement in a variety of fields. In the field of cancer research, PTDOs have shown great promise in the study of cancer biology and drug development. First, it is much faster to establish a PTDO line (weeks for PTDOs rather than months for GETM and PDTX). Second, in comparison with cultured cancer cell lines, PTDOs are more likely to capture the heterogeneity of the parental tumors. Third, since laboratory animals are not needed to establish and maintain the PTDOs, the cost to establish and maintain the PTDOs is much lower than that of GETM and PDTX. Therefore, it is possible to establish and proliferate a sufficient number of PTDO lines for long‐term preservation of patient samples to be later utilized in drug screening.

The studies discussed above detail the utility of organoid cultures in recapitulating the histologic and genetic characteristics of parental tumors of many different types of cancers. Organoids can be generated from a wide variety of neoplasms and survive many passages while maintaining vital characteristics of parental tumors. These models were subjected to screening with various chemotherapeutic and targeted therapy agents, and organoid response often mirrored that of the parental tumor.

On the other hand, more research still needs to be done on the PTDOs. For example, many researchers noted that establishing organoids from low‐grade tumors is often harder than from high‐grade tumors. Developing new biomaterials that more closely mimic the extracellular matrix of different tissues/organs may increase the successful rate of establishing PTDOs from low‐grade tumors. Incorporating blood vessel network may also increase the successful rate of establishing PTDOs from low‐grade tumors. In addition, coculturing PTDOs with patient immune cells will make it possible to test the immuno‐oncology drugs using the PDTOs. Finally, the drug testing results using the PTDOs still need to be further validated using animal models and clinical trials.

**Figure 2:**
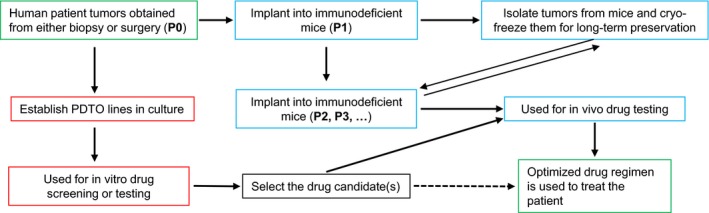
A proposed strategy of using the PDTO to optimize the drug regimen for cancer patients in the clinic

Nonetheless, the prospect of utilizing organoids in cancer drug development is an exciting one. The idea that tumor organoids can be established from tumor biopsies or surgically removed tumor tissues, and then used for prescreening to find optimal treatment regimens for cancer patients (Figure 2), may be realized in the near future.

## CONFLICT OF INTEREST

None.
